# Mapping of subthalamic nucleus using microelectrode recordings during deep brain stimulation

**DOI:** 10.1038/s41598-020-74196-5

**Published:** 2020-11-06

**Authors:** Nabin Koirala, Lucas Serrano, Steffen Paschen, Daniela Falk, Abdul Rauf Anwar, Pradeep Kuravi, Günther Deuschl, Sergiu Groppa, Muthuraman Muthuraman

**Affiliations:** 1 Movement Disorders and Neurostimulation, Biomedical Statistics and Multimodal Signal Processing Unit, Department of Neurology, Johannes Gutenberg University, Mainz, Germany; 2 Department of Neurosurgery, Johannes-Gutenberg-University, Mainz, Germany; 3 Department of Neurology, Christian Albrechts University, Kiel, Germany; 4 Department of Neurosurgery, Christian Albrechts University, Kiel, Germany; 5 Biomedical Engineering Centre, UET Lahore (KSK Campus), Lahore, Pakistan

**Keywords:** Neurodegenerative diseases, Parkinson's disease

## Abstract

Alongside stereotactic magnetic resonance imaging, microelectrode recording (MER) is frequently used during the deep brain stimulation (DBS) surgery for optimal target localization. The aim of this study is to optimize subthalamic nucleus (STN) mapping using MER analytical patterns. 16 patients underwent bilateral STN-DBS. MER was performed simultaneously for 5 microelectrodes in a setting of Ben’s-gun pattern in awake patients. Using spikes and background activity several different parameters and their spectral estimates in various frequency bands including low frequency (2–7 Hz), Alpha (8–12 Hz), Beta (sub-divided as Low_Beta (13–20 Hz) and High_Beta (21–30 Hz)) and Gamma (31 to 49 Hz) were computed. The optimal STN lead placement with the most optimal clinical effect/side-effect ratio accorded to the maximum spike rate in 85% of the implantation. Mean amplitude of background activity in the low beta frequency range was corresponding to right depth in 85% and right location in 94% of the implantation respectively. MER can be used for STN mapping and intraoperative decisions for the implantation of DBS electrode leads with a high accuracy. Spiking and background activity in the beta range are the most promising independent parameters for the delimitation of the proper anatomical site.

## Introduction

Deep brain stimulation of the subthalamic nucleus (STN-DBS), now established as a standard therapeutic option, is an effective therapy for patients with Parkinson’s disease (PD)^1^. The principle effects of the stimulation are for the improvement of major clinical motor symptoms like tremor, rigidity, and bradykinesia^2^. Parkinson’s disease and the effect of DBS for optimal clinical response have been shown to be a network level effect^3,4^. However, both clinical and computational observations have shown that the success of STN-DBS depends fundamentally in placing the DBS electrodes with high precision into the sensorimotor region of the STN corresponding to the dorsolateral posterior part of the nucleus^5–9^. The somatotopic arrangement of sensorimotor region in subthalamic nucleus and its relation to movement and tremor in PD patients has been well established^10,11^. To accomplish a high precision implantation in this region, preoperative MRI images-based navigation systems and intraoperative microelectrode recordings (MER) are widely used. The visualization technique for locating the STN by using preoperative MRI is prone to giving inaccurate results due to brain shift induced parenchymal alterations or cerebrospinal fluid loss^12^. Hence, the intraoperative MER allows improving the target localization during stereotactic surgery by recording the electrical activity of the individual neurons from targeted structure. The main principle of this procedure for the STN-DBS is based on the fact that spike patterns for neurons in the subthalamic nucleus (STN) are characteristic and differ from neuronal spike patterns of the surrounding structures^13^. Several previous studies have shown the association of dorsolateral posterior STN field potentials and firing rate to effective clinical outcome^13–15^, quantification of motor subtypes^16^, severity of rigidity and bradykinesia in PD^17^, optimal DBS implantation trajectory^18^ and simulation parameters^19^ among others. Recent studies have further used various recordings using MER for localizing dorsal–ventral border of STN^20^ and predicting therapeutic volume of tissue activation^21^. Despite extensive research in the pathophysiological role and clinical correlation of these electrophysiological activity patterns in PD and target localization, studies focusing on the differential use of them for improving the accurate implantation of the electrodes in sensorimotor region of STN is still insufficient. Moreover, specific use of all MER parameters either to accurately target the STN region or to characterize any clinically relevant outcome has not been thoroughly investigated. In previous studies, it has been reported that most probable track of MER needle will pass through thalamus, zona incerta (ZI), STN and SNr in the same order. During which Thalamus and ZI could be characterized by low firing rates (average number of spikes) along with low amplitude of background activity but in case of STN, both firing rate and amplitude of background activity is reported to be high. Even though SNr exhibits different frequencies and shapes of spikes compared to STN, the relatively high firing rates and very close proximity to STN complicates the task of differentiating the two structures during surgery based only in firing rates^17^. Moreover, SNr has been proven to be promising target for deep brain stimulation (DBS) to treat the gait and postural disturbances in Parkinson’s disease (PD)^22,23^ but the critical development of differentiating STN and SNr border and positioning the DBS electrode within the SNr is still being investigated in recent studies^24,25^. In this study, we investigate extensive list of parameters using both spikes and background activity obtained using MER in different depth and locations in both STN and SNr for localizing optimal target location and depth. Here in this study, we show that by computing data driven parameters like maximum spike rate and beta frequency amplitude, we could accurately differentiate STN and SNr for achieving optimal therapeutic benefit while avoiding complications. We propose this method to exploit the obtained characteristics of spikes and background activity in different brain regions to complement the MRI-based detection of the sensorimotor region of STN and precisely predict the target location and depth in PD patients during DBS surgery.

## Methods

### Subjects and surgical procedure

Sixteen patients with idiopathic PD without dementia (12 males, age 64.06 ± 7.68, Hoehn and Yahr (H &Y) 3.19 ± 0.66) selected for DBS treatment were included in this study. All patients underwent bilateral STN implantation as previously described^26^. STN was targeted by stereotactic magnetic resonance imaging and microelectrode recording. As a part of the clinical routine, all patients were tested intraoperatively by a neurologist for the most common symptoms of overstimulation or stimulation outside of the target. They vary from disturbances of speech, eyelid apraxia, mydriasis, hypersalivation, diplopia, rigidity and verbal fluency. The permanent electrode (model 3389 DBS, Medtronic plc, Dublin, Ireland) and pulse generators (Activa Medtronic, biphasic stimulation) were implanted. The pulse setting was 60 μsec in duration at 130 Hz, with voltage adjusted to the individual patient. The study protocol was done in accord with the ethical standards in accord with the Helsinki Declaration of 1975 and were approved by local ethics committee (Ethik commission, medizinische fakueltaet der Christian-Albrechts-Universitaet zu Kiel) and all patients gave the written informed consent for their anonymized data to be used for the research.

### MER data acquisition

The MER data (collected using Lead point MER acquisition system from Medtronic plc, Dublin, Ireland) from patients was recorded from all electrodes (for each side separately) inserted in either side of brain. Data was recorded simultaneously from all five needles arranged in standard “Ben’s Gun” pattern in each hemisphere for approximately 30 s (mean recording time for the whole group was 42.20 ± 7.43 s) with the sampling rate of 24,000 Hz. Considering the target location at 0 mm, MER recordings were obtained from each position at 1 mm MER displacement steps (depths), 10 mm above the dorsal border and 4 mm below the target nuclei. A microelectrode can detect the changes in the extracellular field caused by the current flows from the closest neuron and from other nearby cells^27^. For extracellular recordings, spikes are commonly identified as voltage signals that exceed a threshold, the details of different types of spikes and their origination has been very well reviewed in^28^.

### MER data analysis

MER data was analysed offline using in-house customised MATLAB (Mathworks Inc., Natick, USA) script and wave_clus (a MATLAB package for spike detection)^29^. The MER data and the analysis was segregated into following three components to extract different parameters of interest^30^.

- Artifacts removal

Here, artifacts are defined as the unwanted noise resulting from interference from power line, surgical equipment and other exogenous noises. The recorded MER data was trimmed to 30 s length for all the participants and was subjected to segmentation for removing exogenous artifacts. Segmentation is a process of dividing the data into epochs—a process of extracting specific time-windows from the continuous signal. Individual epochs were then inspected for the presence of artifacts and were discarded. In order to avoid artifacts due to concatenation of non-adjacent epochs we interpolated the epochs with pchip (Piecewise Cubic Hermite Interpolating Polynomial) method^31^. During segmentation, the MER data was divided into non-overlapping epochs of 0.5 s and was arranged in an array S = [s(1), s(2),…, s(l)], where [s(1), s(2), …, s(l)] are non-overlapping epochs of MER data vector S. Afterwards, the variance coefficient (V_c_) for each epoch was calculated by computing the quotient of variance using the following expression:

1 $${V}_{c}= \frac{var\left\{s\left[k\right]\right\}}{var\left\{s\left[k-1\right]\right\}}$$

A critical threshold of 1.8 was set and all the epochs with value of V_c_ greater than this were discarded from further analysis^32^. Thus, obtained clean epochs were then analyzed individually for background activity and spike detection.

- Spikes detection

**Figure 1:**
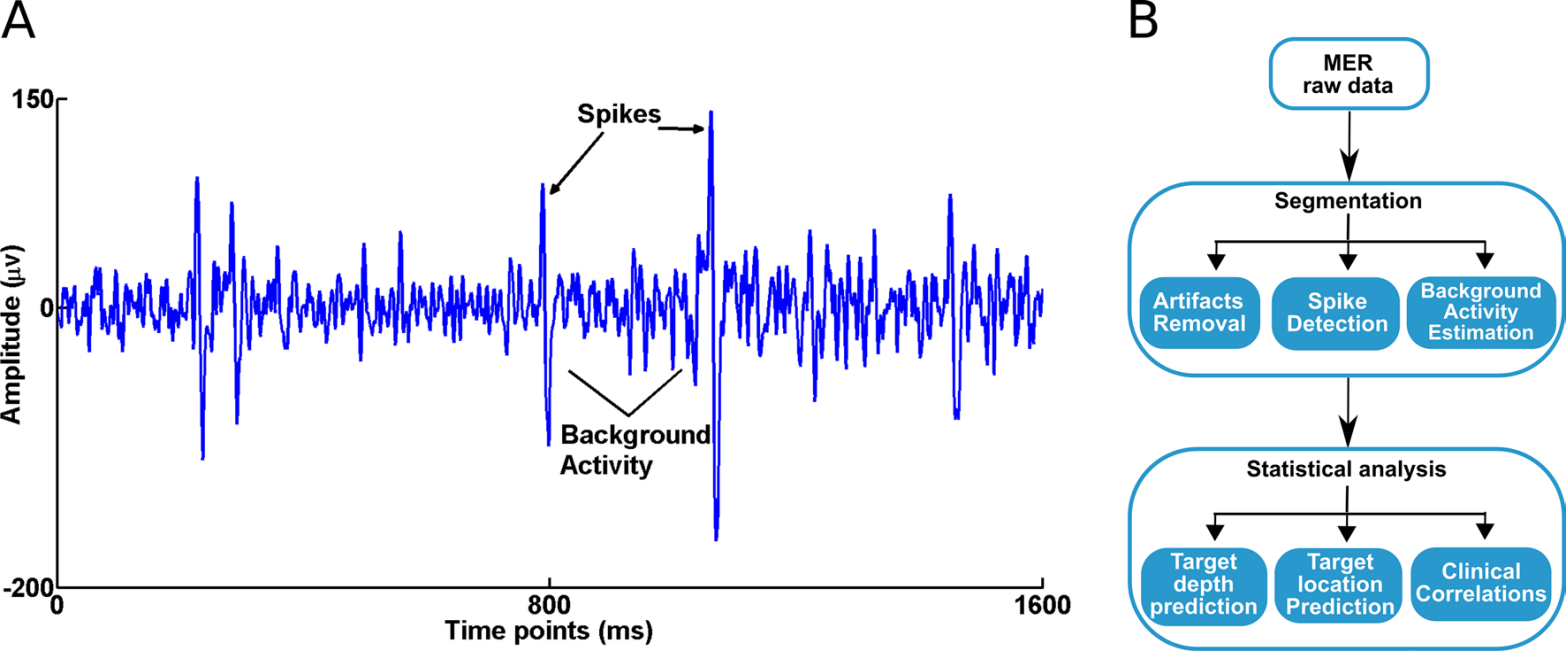
(**A**) Example of the raw MER data indicated with spikes and the background activity. (**B**) The pipeline for processing of the MER data.

The algorithm used for spike detection could be divided into three main steps—spike sorting and detection, selection of spike features and clustering of selected spike features. The details of this method have been explained in detail previously in^32,33^, but to mention here briefly. First, a band pass filter (500 and 5000 Hz using fourth order Butterworth filter) was used to filter the high power, low frequency activity in order to visualize the spikes. One of the distinct features for classification of the background activity and spikes are the signal amplitude. Typically, the amplitude of background activity ranges from 2 to 6 µV whereas the typical spike amplitude ranges from 70 to 150 µV (Fig. 1A). Here, we consider multi-unit spiking activity to be the signal from putative action potential.

In the first step, after removing the exogenous artifacts, spikes are detected with an automatic amplitude threshold. In the second step, wavelet coefficients from each spike was calculated using a four-level multi-resolution decomposition using Haar wavelets and 64 wavelet coefficients were obtained for each spike. Each wavelet coefficient here would characterize the spike shapes at different scales and times. Hence, to choose the coefficients that best distinguishes different spike shapes, Lilliefors modification of Kolmogorov–Smirnov (KS) test for normality was used^34^. These selected coefficients representing a compressed set of spike features are then used as the input for clustering algorithm. In the final step, superparamagnetic clustering (SPC) was used for unsupervised clustering of the spikes^35^. SPC is based on the concept of simulated interactions between each data points and its K-nearest neighbors carried out in two steps. In the first step, interaction strength is computed (using Eq. 2) between each feature (similar spikes will have strong interaction) and in the second step Monte Carlo iterations (here 500) is performed for wolf algorithm to compute the probability of the change in state of nearest neighbors for different temperature (using Eq. 3)^36,37^.

2 $${J}_{ij}= \frac{1}{K}\mathrm{exp}\left(-\frac{\Vert {x}_{i}- {x}_{j}\Vert { }^{2}}{{2a}^{2}}\right)$$

Here, strength of interaction ${J}_{ij}$ is as above if ${x}_{i}$ is a nearest neighbor of ${x}_{j}$, else 0. For the equation, *a* is the average nearest-neighbors distance and *K* is the number of nearest neighbors.

3 $${p}_{ij}=1-exp\left(-\frac{{J}_{ij}}{T}{\delta }_{si,sj}\right)$$

Here, ${p}_{ij}$ is the probability that the nearest neighbor of ${x}_{i}$ will also change their state. T is temperature and ${\delta }_{si,sj}$ is the point-point correlation whose details is discussed in^38^.

The clusters are thus formed based on the principle that, for temperatures corresponding to the superparamagnetic phase, only those points that are grouped together will change their state simultaneously^38^. A brief schematic workflow of spike detection and classification is shown in Fig. 1B.

- Background activity

Here we consider background activity to be the electrical activity of other cells around the recording electrode which could range from action potential from distant cells to subthreshold events in neurites of nearby cells or synaptic noise caused by the stochastic synaptic transmission^39^. After spike sorting, detected spikes with shapes and timestamps are saved, these timestamps are used for reconstruction of background noise. The reconstruction is done by substituting 0.5 ms before to 2.5 ms after each timestamp with 3 ms of spike-free consecutive signal from a random location within the same recorded trace. The reconstructed background noise was passed through a low pas filter of 500 Hz and is left with no significant spikes, leaving only a few, near-noise level and secondary neuron’s spikes^35^.

- Parameters computation

After the segmentation of spikes and background activity, overall mean for both of these parameters was estimated for each subject separately. The estimation was done quantitatively by calculating the difference in variance for every 0.5 s to identify the artifact-prone segments. Using the spikes, modal interspike interval (ISI) and burst index (BI) were further assessed. For computing ISI, the most frequent value in the distribution (mode) was considered for the estimation. However, analysis of raw ISI has been shown to be vulnerable to fluctuations in the firing rate and an improved measure to incorporate these irregularities have been suggested before^40–42^. Hence, we further computed Local variation compensate for Refractoriness (LvR) (using Eq. 4) which has been shown to measure the local variation of ISI and enhance the invariance to firing rate fluctuations increasing the sensitivity of detection of signal characteristic^40,43^.

4 $$LvR= \frac{3}{n-1}\sum_{i=1}^{n-1}\left(1- \frac{4{I}_{i}{I}_{i+1}}{{\left({I}_{i}+{I}_{i+1}\right)}^{2}}\right)\left(1+ \frac{4R}{({I}_{i }+ {I}_{i+1})}\right)$$

Here, ${I}_{i}$ and ${I}_{i+1}$ are the i^th^ and i + 1^th^ ISIs, and n is the number of ISIs. R is the refractoriness constant.

Moreover, Burst index (BI) was computed as the reciprocal of the modal interval divided by the mean firing rate^44^. This index is computed based on the principle of maximization of Poisson surprise, whose detail derivation and formulation is explained in^45^.

Using the background activity, we further computed the parameters to observe the oscillatory characteristics of the obtained signal. Previous studies have revealed that basal ganglia activity in Parkinson's disease involves increase in firing rate, a tendency toward bursting and abnormal synchronization in the neurons of the subthalamic nucleus (STN)^46–48^. These abnormalities and association to the outcome of DBS has been observed in various frequency bands^49–51^. Hence, we computed spectral estimations (mean amplitude, maximum peak and root-mean-square value) in a range of frequency bands—low frequency (2–7 Hz), Alpha (8–12 Hz), Beta (sub-divided as Low_Beta (13–20 Hz) and High_Beta (21–30 Hz) ) and Gamma (31 to 49 Hz) using the power spectrum density with Welch method^35^.

### Target localization and evaluation

During the surgery, coordinates for anterior commissure (AC) and posterior commissure (PC) are determined and adapted to the coordinates of STN by direct visualization, which is in general 12 mm lateral, 3 mm posterior and 3 mm caudal in relation to Mid-AC-PC. Hence determined individualized depth is considered to be the correct target depth in the study. Furthermore, dorsolateral region of STN (individually determined for each subject using clinical/surgical procedure) is the target location and is considered as the correct implantation site. The optimal target location and depth for the electrodes were further verified by evaluating the Unified Parkinson’s disease rating scale (UPDRS) score pre-operative (one week before) and post-operative (three months after). Only those patients were considered in the study who were significantly improved (UPDRS improvement of 75% or more, with medication) after the surgery.

All parameters mentioned above were calculated for the MER data recorded at different depths (from 10 mm above and 4 mm below the target nuclei). These parameters were then used for the prediction of implantation site (location) and correct depth and for the comparison with the results from visual inspection. So, the underlying hypothesis being, depths at which these parameters are observed to have relatively high amplitudes compared to other depths, resembling the visual inspection and is the target stimulation location in STN. Hence, the detection accuracy is calculated based on the number of times obtained parameters maximum value and clinical target coincides. Here, an experienced Neurologist (SF) specialized in movement disorders also observed the spike rate visually (VSR) for the purpose of comparison. Additionally, the parameters obtained in the target location were further correlated with clinical parameters using Pearson correlation coefficient for determining the sensorimotor subdivision of the STN. The multiple comparison correction was performed for all the correlation results for each subset of independent variables using Bonferroni correction.

## Results

32 possible final target locations from 16 subjects with bi-lateral DBS electrodes were identified. Mean Fourier transformed Beta band amplitude from the background activity in STN corresponded to the target depth in 81.25% of the subjects. When splitting beta in low and higher frequency bands, lower beta (13–20 Hz) was accord to the target depth with even greater accuracy in 84.37% of the subjects. Mean Gamma band amplitude computed using the background activity in SNr corresponded to the target depth in 85.71% of the subjects. Similarly, the spikes rate in STN was in accord to the target depth in 84.37% of the subjects and the number of spikes in SNr in 100% of them. Among other parameters, LvR was in accord to target depth precisely in 78.12% of the subjects. Table 1 shows the details of all computed parameters using the background activity, spikes, interspike interval and burst index and prediction accuracy.

**Table 1:** Successful target depth detections using background activity and spikes.

Parameters	STN, successful detection	SNr, successful detection
Mean_low_freq	56.25% (18/32)	14.28% (2/14)
Max_low_freq	59.37% (19/32)	15.38% (2/13)
Mean_Alpha	62.50% (20/32)	25.00% (3/12)
Max_Alpha	65.62% (21/32)	27.27% (3/11)
Mean_low_Beta	84.37% (27/32)	40.00% (2/5)
Max_low_Beta	78.12% (25/32)	28.57% (2/7)
Mean_high_Beta	71.87% (23/32)	33.33% (3/9)
Max_high_Beta	68.75% (22/32)	30.00% (3/10)
Mean_Beta	81.25% (26/32)	16.67% (1/6)
Max_Beta	75.00% (24/32)	25.00% (2/8)
Mean_Gamma	78.13% (25/32)	85.71% (6/7)
Max_Gamma	68.75% (22/32)	80.00% (8/10)
Mean_RMS	50.00% (16/32)	18.75% (3/16)
Max_RMS	75.00% (24/32)	25.00% (2/8)
ISI	68.75% (22/32)	25.00% (2/8)
BI	75.00% (24/32)	50.00% (4/8)
LvR	78.12% (25/32)	28.57% (2/7)
Mean_SR	53.12% (17/32)	73.33% (11/15)
Max_SR	84.35% (27/32)	100% (5/5)
Mean_VSR	34.38% (11/32)	9.52% (2/21)
Max_VSR	6.25% (2/32)	13.33% (4/30)

The number of successful target depth detections using individual parameters extracted from the Background activity and spikes in the STN and SNr. Mean_low_freq and Max_low_freq, Mean and Maximum amplitude respectively in low frequency range (2–7 Hz); Mean_alpha and Max_alpha, Mean and Maximum amplitude respectively in alpha frequency range (7–13 Hz); Mean_low_Beta and Max_low_Beta, Mean and Maximum amplitude respectively in lower beta frequency range (13–20 Hz); Mean_high_Beta and Max_high_Beta, Mean and Maximum amplitude respectively in higher beta frequency range (21–30 Hz); Mean_Beta and Max_Beta, Mean and Maximum amplitude respectively in beta frequency range (13–30 Hz); Mean_Gamma and Max_Gamma, Mean and Maximum amplitude respectively in gamma frequency range (31–49 Hz); Mean_RMS and Max_RMS, Mean and Maximum root mean square amplitude respectively of the background activity; ISI, Interspike interval; BI, Burst index and LvR, Local variation compensate for Refractoriness; Mean_SR and Max_SR, Mean and Maximum spiking rate respectively; Mean_VSR and Max_VSR, Mean and Maximum visual spiking rate respectively.

**Figure 2:**
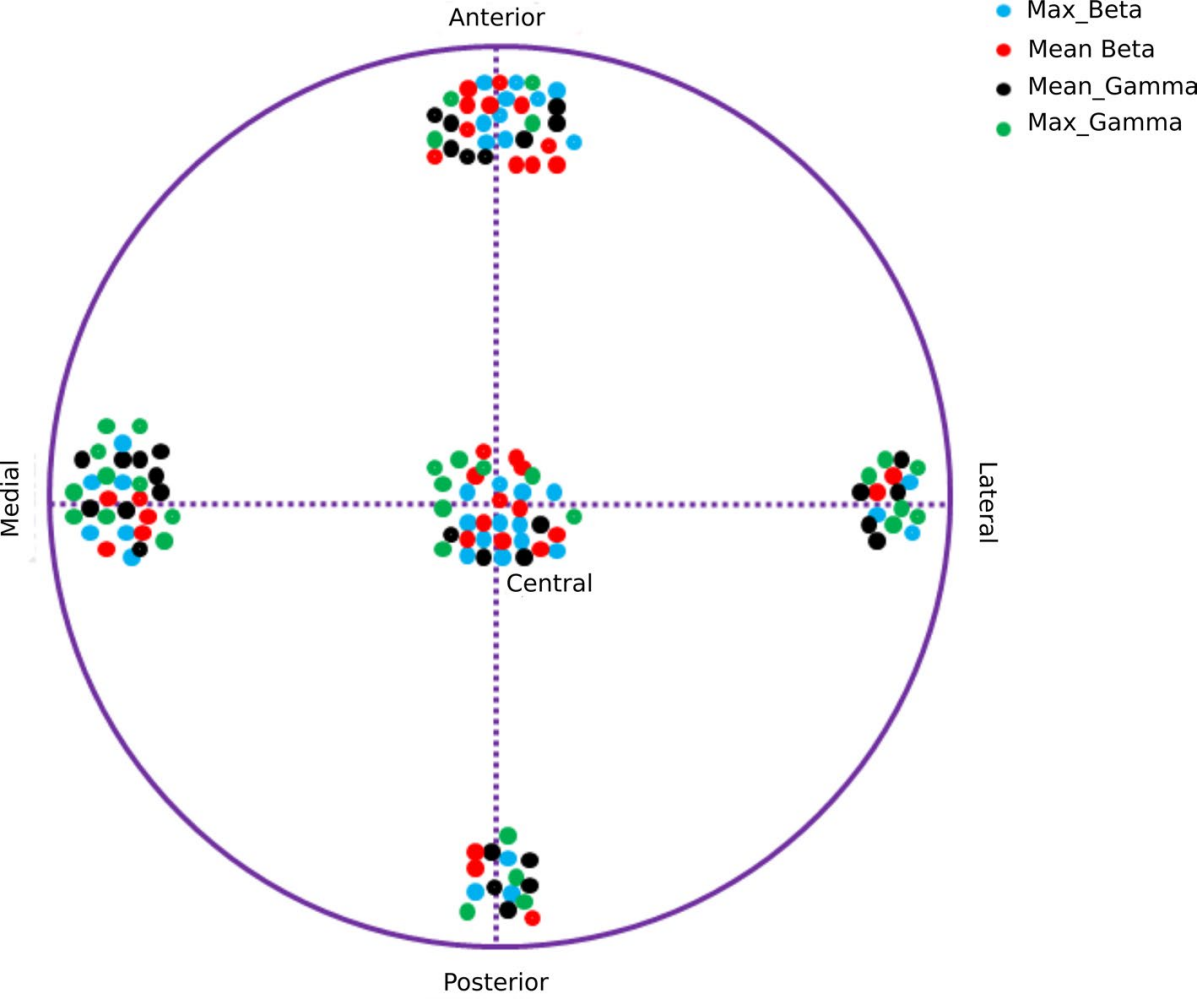
Target location detected correctly from five different MER recording trajectory using the four parameters (Mean_Beta, Mean amplitude in beta frequency range (13–30 Hz); Max_Beta, Maximum amplitude in beta frequency range; Mean_Gamma, Mean amplitude in gamma frequency range (31–49 Hz); Max_Gamma, Maximum amplitude in gamma frequency range are depicted.

**Figure 3:**
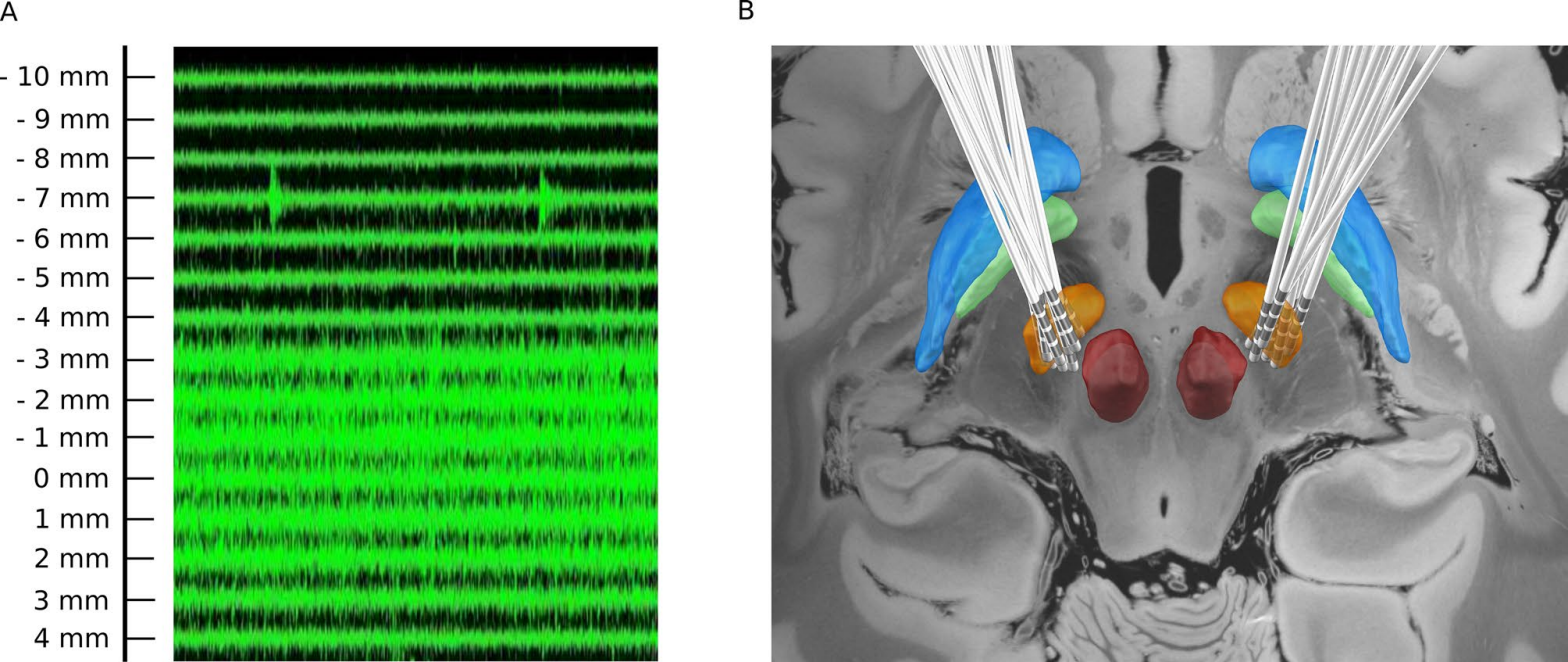
(**A**) Raw microelectrode recordings (MER) obtained from different depth above and below the target. (**B**) Final electrode positions in all patients, using Lead-DBS 2.0 (https://www.lead-dbs.org/). Subcortical structures are based on DISTAL atlas (Orange, STN; Green, GPi; Blue, GPe and red, red nucleus) and the background template is 7 T MRI ex vivo 100 micron human brain.

**Figure 4:**
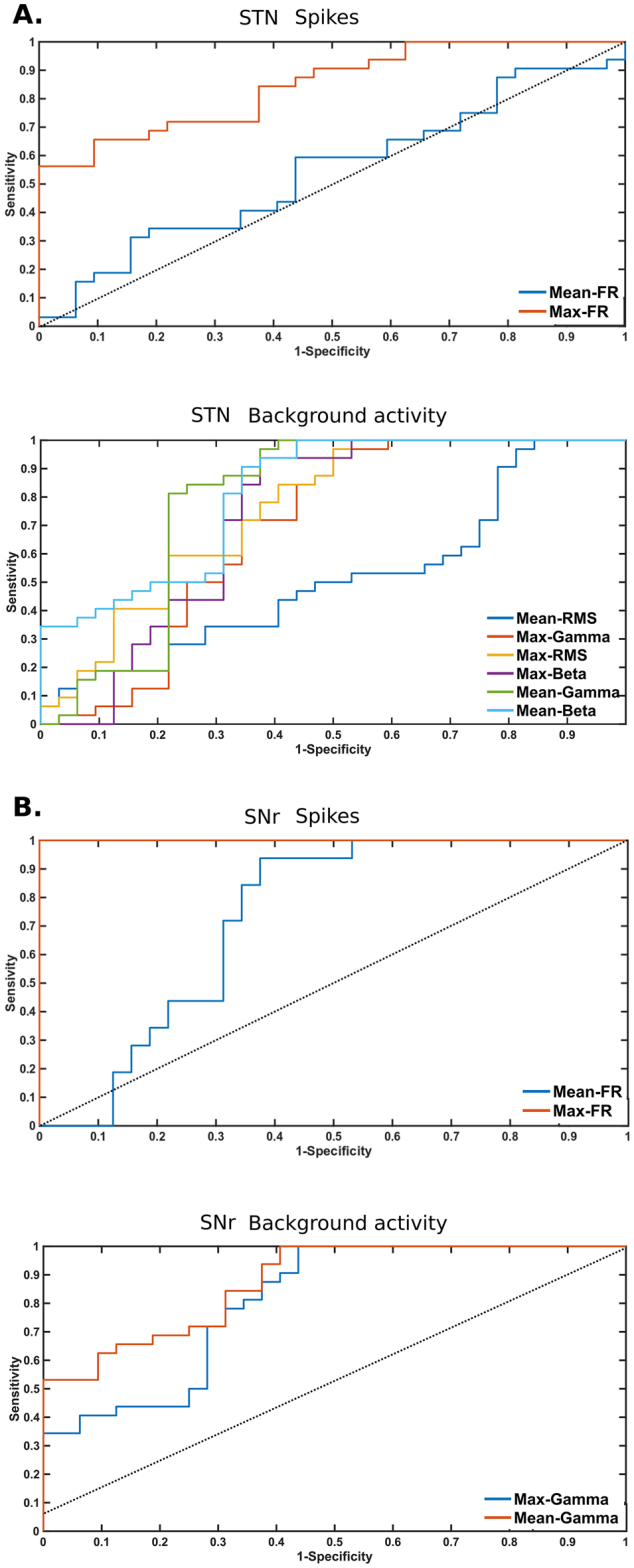
Receiver operating characteristics (ROC) curve for the selected parameters obtained using spikes and background activity in the study. Area under the curve (AUC) with significance p < 0.000001 for Mean_Beta in STN was 0.81250 ± 0.05426 (standard error), Mean_Gamma in SNr was 0.84668 ± 0.04951, Max_SR in STN was 0.84328 ± 0.04653 and in SNr was 1.0.

For the localization of target location, maximum amplitude from lower beta frequency was found to have the best accuracy, corresponding in 93.75% of the subjects. Moreover, mean of low beta band was only corresponding for 84.7% but the using overall beta band (13–30 Hz) the accuracy increased to 87.5% of the subjects. Mean and maximum amplitude in gamma frequency could only correspond to 68.75% and 65.60% of the subjects respectively. However, maximum spike rate was corresponding to the target location in 87.5% of the subjects. Details of all computed parameters and prediction accuracy for target location is shown in Table 2. Mean beta misclassified four target points (two from the central and lateral respectively) as two from posterior and anterior respectively. The maximum beta amplitude misclassified only two target points one from the central and one from the medial. The mean gamma amplitude misclassified ten target points (five from the anterior and central respectively) as five from lateral and medial respectively. The maximum gamma amplitude misclassified only 11 target points, eight from the central and three from the anterior. The correct prediction of the target location with mean and maximum amplitude of beta and gamma is as shown in Fig. 2. The schematic of the actual location of the electrodes are depicted in Fig. 3A and the reconstruction of the implanted electrodes using LeadDBS^52^ with projection to the MNI anatomical T1 template in Fig. 3B. In addition, receiver operating characteristics (ROC) curve results of the primary findings presented in the study is as shown in Fig. 4.

**Table 2:** Successful detections of target location using background activity and spikes**.**

Parameter	Successful detection (STN)
Mean_low_freq	50.00% (16/32)
Max_low_freq	43.75% (14/32)
Mean_Alpha	56.25% (18/32)
Max_Alpha	59.37% (19/32)
Mean_low_Beta	84.37% (27/32)
Max_low_Beta	93.75% (30/32)
Mean_high_Beta	65.62% (21/32)
Max_high_Beta	56.25% (18/32)
Mean_Beta	87.50% (28/32)
Max_Beta	93.75% (30/32)
Mean_Gamma	68.75% (22/32)
Max_Gamma	65.60% (21/32)
Mean_RMS	75.00% (24/32)
Max_RMS	56.25% (18/32)
ISI	62.50% (20/32)
BI	71.87% (23/32)
LvR	78.12% (25/32)
Mean_SR	81.25% (26/32)
Max_SR	87.50% (28/32)
Mean_VSR	65.62% (21/32)
Max_VSR	68.75% (22/32)

The number of successful detections of target location (STN) using individual parameters obtained from Background activity and spikes. Mean_low_freq and Max_low_freq, Mean and Maximum amplitude respectively in low frequency range (2–7 Hz); Mean_alpha and Max_alpha, Mean and Maximum amplitude respectively in alpha frequency range (8–12 Hz); Mean_low_Beta and Max_low_Beta, Mean and Maximum amplitude respectively in lower beta frequency range (13–20 Hz); Mean_high_Beta and Max_high_Beta, Mean and Maximum amplitude respectively in higher beta frequency range (21–30 Hz); Mean_Beta and Max_Beta, Mean and Maximum amplitude respectively in beta frequency range (13–30 Hz); Mean_Gamma and Max_Gamma, Mean and Maximum amplitude respectively in gamma frequency range (31–49 Hz); Mean_RMS and Max_RMS, Mean and Maximum root mean square amplitude respectively of the background activity; ISI, Interspike interval; BI, Burst index and LvR, Local variation compensate for Refractoriness; Mean_SR and Max_SR, Mean and Maximum spiking rate respectively; Mean_VSR and Max_VSR, Mean and Maximum visual spiking rate respectively.

Further, a significant positive correlation was observed in STN between the maximum spike rate and maximum overall beta frequency amplitude (r = 0.709, p = 0.0002) which is probably driven by the lower beta band as the correlation of it to maximum spike rate was significant (r = 0.649, p = 0.0009) which was not the case for high beta band (r = 0.208, p = 0.30). Moreover, a significant correlation between maximum overall beta frequency amplitude and LvR was observed (r = 0.487, p = 0.007), similarly swayed by the correlation to low beta frequency band (r = 0.576, p = 0.008). A negative correlation between the mean amplitude in the gamma frequency band to maximum beta (r = -0.603, p = 0.0013) and to mean of low beta (r = − 0.575, p = 0.004) was further observed in STN. For clinical associations, correlations to the pre-operative UPDRS scores to the maximum beta frequency amplitude in STN also yielded significant results [Med off: r = 0.767, p = 0.000005, Med on: r = 0.602, p = 0.0013]. The percentage of levodopa reduction after the surgery was also found to be negatively correlated with the mean gamma amplitude in STN [r = − 0.579, p = 0.0024]. All correlation results have been detailed in supplementary table.

## Discussion

When comparing the parameters characterizing spike from MER recordings of STN targeting, our results revealed that maximal spiking rate provided the highest accuracy for the localization of the sensorimotor region of the STN in PD patients. Similarly, for those parameters obtained from background activity, mean low beta amplitude offered the highest accuracy for both target depth and target localization.

The most movement-related cells are located anterodorsally in STN and is the most effective target for high frequency stimulation in terms of clinical benefits for PD^53^. This somatotopic organization within the nucleus (sensorimotor, limbic and associative) has been further supported by recent high-resolution neuroimaging studies^54^. Moreover, Parkinson’s disease has been associated with the enlarged receptive fields in PD patients in comparison to healthy controls resulting in increased perceptive thresholds, reduced discriminative capacities and a mildly increased firing rate with bursting activity^55,56^. The STN, constituted mostly by excitatory glutamatergic neurons normally fires in an irregular pattern at medium frequency rates between 25 and 45 Hz^57^. In PD, degeneration of dopaminergic neurons located into the substantia nigra pars compacta (SNc) results in an hyperactivity of the STN and therefore an increase of its firing rates^17^. This fact also has been supported by experimental evidence utilizing the MPTP model of experimental parkinsonism in monkeys^48,58,59^. Even though increased firing rate of the STN is in this aspect a consistent finding in PD, the identification of the sensorimotor region of the STN based only on MER recordings presents technical limitations. During STN-DBS surgery, after the microelectrode passes in its trajectory through the dorsal region of the STN, it reaches a thin white matter layer before entering into the substantia nigra pars reticulata (SNr). This white matter layer is only a few hundred microns thick and usually cannot be identified before the electrode reaches the SNr. In addition, the firing characteristics (reduced β band and tremor frequency oscillations) in the cells of STN ventral domain resembles the firing characteristics of SNr cells^24^. Although recent imaging studies have markedly contributed for improving the localization of STN or SNr regions , the intraoperative distinction of electrode placement within the dorsal STN or the SNr based on firing rate is still a daunting task. Hence, the obtained results of maximal firing rates from different depth and location detecting both the STN and SNr accurately supports previous findings and further facilitates a simpler and accurate technique for target distinction during the surgery.

In recent years, enhanced beta band activity in STN has been revealed in both animal model and humans for Parkinson’s disease^60–62^. Several studies have demonstrated high amplitudes of beta-activity inside the dorsolateral portion, i.e. sensorimotor region, of the STN in PD patients^63–65^ and stimulation of this region produces the greatest improvement in parkinsonian motor signs^15,63,66–69^ and distinction of these region could even be used to identify the physiological signatures of PD subtypes^16,70,71^. Increased beta band activity shows a remarkable selectivity for neuronal clusters connected directly with the primary motor cortex on the dorsolateral portion of the STN and decreases medially towards the associative sub region of the STN, where most projections are from the premotor cortices to enhance the alpha band activity^14,72,73^. These studies demonstrate that in addition to the spiking rate the increased beta band activity is also an important factor for determining the correct target with MER recording. We found in our study that spiking rate correlated directly with beta band activity into the STN and both mean and maximal beta band amplitude particularly that in the lower frequency band (13–20 Hz) were highly accurate to identify the dorsolateral region of the STN in PD patients. These results hence validate the notion that high beta activity can be used to functionally isolate the sensorimotor division of the STN from other regions in PD patients using MER recordings. Moreover, the lack of localization accuracy (all lower than 70%) from lower frequency bands (low_freq and alpha) might further supports the findings of beta band oscillations having different temporal and spatial properties than these oscillations and is uncorrelated to tremor related oscillations^35,74,75^. It has been shown previously that increase of beta frequency within the STN correlates with the severity of motor disturbances in PD patients, predominantly rigidity and bradykinesia. The reduction of which is observed under treatment with L-Dopa as well as during STN-DBS indicating the significant improvement of motor symptoms^76–78^. We found a strong correlation between maximal beta band activity and worse motor performance in pre-operative UPDRS scores during both ON and OFF medication states. This suggests that the identification of the sensorimotor subdivision of the STN based on increased beta band activity may be of maximal accuracy in the subgroup of patients with severe rigidity and bradykinesia. However, when separating beta in low and high frequency band, the correlation to UPDRS score during both medication ON and OFF states were different with maximum amplitude of low_beta significantly correlating to UPDRS but not the maximum amplitude of high_beta band. This discrepancy might be linked to previously postulated theories of beta activity in human STN having an ‘antikinetic’ role which could be more specifically played by the low-beta rhythm, whereas the high-beta rhythm could be essentially physiological^46,50,61^.

Along with beta band, gamma band activity into the STN in PD patients has also been shown to be directly generated within the STN and might play a pro kinetic role^79–82^. Gamma oscillations have also been shown to be increased during imagination and voluntary performance of grips and movements^83^, promoting movement by cortical stimulation to increases the rate of force production^80^ and as well correlates with improvement of motor symptoms after treatment with L-Dopa in PD^84^. In contrast to the beta band activity, oscillatory field potential activity in PD patients during off medication is less prominent in the gamma band, existing a negative correlation between beta and gamma oscillations in STN after treatment with dopaminergic medications^85^. In line with these findings, we observed a negative correlation between the mean gamma and maximal beta band activity. However recent publications have shown that the coupling between beta and gamma activity is pathological^86^. Some of them argues that beta phase and gamma amplitude is synchronized via phase-amplitude coupling (PAC) in Parkinson's disease patients and this coupling decrease during DBS^87,88^. But others showed that these pathological coupling between beta and broadband gamma found previously is unlikely to reflect alterations in neural activities at gamma frequencies but rather just the alterations in beta frequency in PD patients^89^. To this regard, although we found a strong negative correlation between maximal beta and mean gamma activity in the STN, there was also a higher reduction of dopaminergic medication after DBS associated to lower mean gamma band activity. Moreover, the presence of high gamma activity in patients with high dose of L-Dopa even after DBS implies an association between gamma oscillations and worse response to DBS treatment. Low-gamma frequency has been also reported to be enhanced during periods of significant rest tremor^90^ and reduction of tremor is associated with gamma power suppression into the STN in patients with PD^91^. It is further suggested that neurons in dorsolateral STN receives inputs in both beta and gamma frequencies and oscillates at gamma frequency during tremor and beta during bradykinesia and rigidity^90^. If the same group of neurons in the STN oscillate at gamma or beta frequencies according to the preponderance of tremor or bradykinesia, it would be expected to have high correlations on mean gamma and mean beta firing activity in our findings for correct target detection. However, from our data we could not directly infer whether there is a correlation between correct target identification through either mean gamma or beta activity and the differential symptomatic pattern of the patient (predominance of tremor vs rigidity/bradykinesia).

The choice of optimal DBS contact for programming has a significant impact on the therapeutic efficacy^13,65^. Moreover, even with the possibility of MER recordings, the localization of the STN and implantation in the right part is still hard to achieve because of coarse spatial resolution of the lead which in turn leads to pick up also electrical activity of areas more remotely located^92^. Hence, the proposed comprehensive set of parameters in this study obtained using both spikes and background activity data in different depths and locations in and outside of STN, we believe would assist the neurosurgeon and neurologist involved in identifying the target with the optimal clinical outcome for the patient with more ease. Even though the MER algorithm is robust enough in detecting the physiologically defined optimal target, cautions should be made in interpretation of the data. As explained in previous literature, with the commercially available systems now having five microelectrodes arranged in a cross with a microelectrode at the end of each segment and one at the center, there would be a substantial probability that the physiologically defined optimal target would not be within the area covered by the fixed microelectrode array^12^.

## Conclusions

The analysis of specific electrophysiological parameters using spike and background activity characteristics from the MER recordings during STN-DBS allows a high accuracy for correct target detection in PD patients. The intraoperative recording and automated analysis of spiking and background activity in the beta range could evolve to a very promising marker with a translational value for the clinical practice. We found that spiking rate was consistent for detecting the target depth and increased beta activity allowed identifying the STN borders and differentiate it from neighboring structures particularly the SNr. Also, the target selection and definitive electrode placement within the STN based on higher beta range activity correlates with an improved clinical outcome and especially an improvement of bradykinesia.

## Supplementary information

Supplementary file1
