# Supplementary material for: Mapping of subthalamic nucleus using microelectrode recordings during deep brain stimulation
Source: Sci Rep. 2020 Nov 6;10:19241. doi: 10.1038/s41598-020-74196-5 (PMC7648837; doi:10.1038/s41598-020-74196-5)
Supplement: Supplementary file 1 — Supplementary file1 [file 41598_2020_74196_MOESM1_ESM.pdf]

Mapping of subthalamic nucleus using microelectrode recordings during deep brain stimulation

Nabin Koirala, Lucas Serrano, Steffen Paschen, Daniela Falk, Abdul Rauf Anwar, Pradeep Kuravi, Günther Deuschl, Sergiu Groppa, Muthuraman Muthuraman.

Supplementary materials

Table 1: The correlation coefficient and P values are listed for the spikes related parameters.

Parameters	Correlation coefficient	P value
Mean_Low Vs Mean_SR	0.21294474	0.26043626
Max_Low Vs Mean_SR	0.23115839	0.61340094
Mean_alpha Vs Mean_SR	0.07539736	0.57556569
Max_alpha Vs Mean_SR	0.23267517	0.38009381
Mean_low beta Vs Mean_SR	0.17647185	0.42348505
Max_Low beta Vs Mean_SR	0.06950808	0.40222682
Mean_high beta Vs Mean_SR	0.10569964	0.17464538
Max_high beta Vs Mean_SR	0.1593763	0.2307478
Mean_beta Vs Mean_SR	0.24150137	0.33255401
Max_beta Vs Mean_SR	0.24297771	0.1882929
Mean_gamma Vs Mean_SR	0.08152262	0.55658528
Max_gamma Vs Mean_SR	0.24411856	0.16685857

Parameters	Correlation coefficient	P value
Mean_Low Vs Max_SR	0.24143339	0.18555307
Max_Low Vs Max_SR	0.14707513	0.15242483
Mean_alpha Vs Max_SR	0.21005609	0.18659858
Max_alpha Vs Max_SR	0.07837727	0.31141921
Mean_low beta Vs Max_SR	0.13435226	0.23666137
Max_Low beta Vs Max_SR	0.64963214	0.00091042*
Mean_high beta Vs Max_SR	0.23314711	0.60402779
Max_high beta Vs Max_SR	0.20844147	0.30812443
Mean_beta Vs Max_SR	0.24189849	0.16088979
Max_beta Vs Max_SR	0.70954642	0.00025455*
Mean_gamma Vs Max_SR	0.18114814	0.59292858
Max_gamma Vs Max_SR	0.05714234	0.63784903

Parameters	Correlation coefficient	P value
Mean_Low Vs Mean_RMS	0.24194879	0.07264332
Max_Low Vs Mean_RMS	0.11807715	0.5811008
Mean_alpha Vs Mean_RMS	0.16705355	0.5979721
Max_alpha Vs Mean_RMS	0.09476239	0.52771032
Mean_low beta Vs Mean_RMS	0.20025341	0.10922737
Max_Low beta Vs Mean_RMS	0.10101902	0.20712271
Mean_high beta Vs Mean_RMS	0.15119141	0.2512141
Max_high beta Vs Mean_RMS	0.18981534	0.45783677
Mean_beta Vs Mean_RMS	0.22818065	0.13193188
Max_beta Vs Mean_RMS	0.24185829	0.4827365
Mean_gamma Vs Mean_RMS	0.15944311	0.11405712
Max_gamma Vs Mean_RMS	0.07772489	0.44225441

Parameters	Correlation coefficient	P value
Mean_Low Vs ISI	0.21982586	0.31332198
Max_Low Vs ISI	0.23679865	0.11667153
Mean_alpha Vs ISI	0.18574703	0.20483882
Max_alpha Vs ISI	0.20154803	0.29523191
Mean_low beta Vs ISI	0.19862649	0.40693764
Max_Low beta Vs ISI	0.1284454	0.20732705
Mean_high beta Vs ISI	0.18109558	0.41170585
Max_high beta Vs ISI	0.08423734	0.47672947
Mean_beta Vs ISI	0.19120922	0.18304804
Max_beta Vs ISI	0.05636657	0.12045059
Mean_gamma Vs ISI	0.1053846	0.22800552
Max_gamma Vs ISI	0.05923428	0.24126698

Parameters	Correlation coefficient	P value
Mean_Low Vs BI	0.06942636	0.30450006
Max_Low Vs BI	0.21469157	0.35471497
Mean_alpha Vs BI	0.18896572	0.10130948
Max_alpha Vs BI	0.1134199	0.20748934
Mean_low beta Vs BI	0.24004441	0.53060877
Max_Low beta Vs BI	0.05688922	0.06753217
Mean_high beta Vs BI	0.13774887	0.60731248
Max_high beta Vs BI	0.12631169	0.48819852
Mean_beta Vs BI	0.20310336	0.34316538
Max_beta Vs BI	0.20903998	0.39711504
Mean_gamma Vs BI	0.08737452	0.19237015
Max_gamma Vs BI	0.14795288	0.3253093

Parameters	Correlation coefficient	P value
Mean_Low Vs Max_RMS	0.0798588	0.34650436
Max_Low Vs Max_RMS	0.10150165	0.51743103
Mean_alpha Vs Max_RMS	0.21814345	0.47902225
Max_alpha Vs Max_RMS	0.10085644	0.59223234
Mean_low beta Vs Max_RMS	0.21285697	0.5845535
Max_Low beta Vs Max_RMS	0.09870499	0.25049783
Mean_high beta Vs Max_RMS	0.23585272	0.4692475
Max_high beta Vs Max_RMS	0.11999675	0.1686859
Mean_beta Vs Max_RMS	0.08931905	0.06832457
Max_beta Vs Max_RMS	0.10021677	0.49644456
Mean_gamma Vs Max_RMS	0.17320894	0.35001346
Max_gamma Vs Max_RMS	0.14465777	0.33795328

Parameters	Correlation coefficient	P value
Mean_Low Vs LvR	0.13911724	0.62785312
Max_Low Vs LvR	0.1792626	0.37808343
Mean_alpha Vs LvR	0.19187297	0.3626815
Max_alpha Vs LvR	0.20093734	0.18895663
Mean_low beta Vs LvR	0.10520502	0.34333865
Max_Low beta Vs LvR	0.57655131	0.00833511*
Mean_high beta Vs LvR	0.18594054	0.42443605
Max_high beta Vs LvR	0.1810196	0.45748132
Mean_beta Vs LvR	0.4874646	0.00715641*
Max_beta Vs LvR	0.08252235	0.28730913
Mean_gamma Vs LvR	0.07379954	0.27046199
Max_gamma Vs LvR	0.14967281	0.6427892

Table 2: The correlation coefficient and P values are listed for the background activity related parameters.

Parameters	Correlation coefficient	P value
Mean_alpha Vs Max_low	0.07133055	0.08628271
Max_alpha Vs Max_low	0.24237962	0.28955466
Max_low beta Vs Max_low	0.05092684	0.3661255
Max_Low beta Vs Max_low	0.20498209	0.30007968
Mean_high beta Vs Max_low	0.21346064	0.44411593
Max_high beta Vs Max_low	0.22373894	0.42678402
Mean_Beta Vs Max_low	0.06688717	0.22519045
Max_ BetaVs Max_low	0.12995653	0.3089907
Mean_Gamma Vs Max_low	0.10197408	0.05929228
Max_Gamma Vs Max_low	0.2100137	0.64043823

Parameters	Correlation coefficient	P value
Mean_alpha Vs Mean_low	0.14010832	0.53019835
Max_alpha Vs Mean_low	0.06676428	0.32227863
Mean_low beta Vs Mean_low	0.09579539	0.3094349
Max_Low beta Vs Mean_low	0.23266747	0.54518828
Mean_high beta Vs Mean_low	0.0804756	0.10008189
Max_high beta Vs Mean_low	0.2151634	0.1299026
Mean_Beta Vs Mean_low	0.15766849	0.15403317
Max_ BetaVs Mean_low	0.24922694	0.28456268
Mean_Gamma Vs Mean_low	0.06563511	0.54882785
Max_Gamma Vs Mean_low	0.13853565	0.53201863

Parameters	Correlation coefficient	P value
Mean_Low Vs Max_Beta	0.05238041	0.07545868
Max_Low Vs Max_Beta	0.11742453	0.09286728
Mean_alpha Vs Max_Beta	0.08243646	0.36298991
Max_alpha Vs Max_Beta	0.20885691	0.10803802
Mean_gamma Vs Max_Beta	0.11224301	0.54088913
Max_gamma Vs Max_Beta	0.15570663	0.54052826
Mean_Low Vs Mean_Beta	0.08312975	0.48346376
Max_Low Vs Mean_Beta	0.17039639	0.13991927
Mean_alpha Vs Mean_Beta	0.10259426	0.44576315
Max_alpha Vs Mean_Beta	0.18081582	0.36115697
Mean_gamma Vs Mean_Beta	0.1878429	0.63378473
Max_gamma Vs Mean_Beta	0.19963032	0.4393949

Parameters	Correlation coefficient	P value
Mean_Low Vs Mean_Gamma	0.06517086	0.34394083
Max_Low Vs Mean_Gamma	0.06079002	0.15075629
Mean_alpha Vs Mean_Gamma	0.15615951	0.63720839
Max_alpha Vs Mean_Gamma	0.20583345	0.47761668
Mean_low beta Vs Mean_Gamma	-0.5746526	0.00475435*
Max_Low beta Vs Mean_Gamma	0.23680214	0.35028297
Mean_high beta Vs Mean_Gamma	0.07598124	0.33265302
Max_high beta Vs Mean_Gamma	0.16376473	0.08577132
Mean_beta Vs Mean_Gamma	0.14387813	0.45918314
Max_beta Vs Mean_Gamma	-0.6035465	0.00131153*

Parameters	Correlation coefficient	P value
Mean_Low Vs Max_Gamma	0.1203319	0.59283334
Max_Low Vs Max_Gamma	0.21616573	0.41591999
Mean_alpha Vs Max_Gamma	0.16705282	0.42059983
Max_alpha Vs Max_Gamma	0.15994472	0.56566538
Mean_low_beta Vs Max_Gamma	0.23343873	0.53329365
Max_Low beta Vs Max_Gamma	0.1071678	0.39603291
Mean_high beta Vs Max_Gamma	0.20144005	0.15975348
Max_high beta Vs Max_Gamma	0.20074582	0.19395921
Mean_beta Vs Max_Gamma	0.12608917	0.58190716
Max_beta Vs Max_Gamma	0.16356433	0.06720449

Parameters	Correlation coefficient	P value
Mean_low Vs Mean_alpha	0.13628277	0.15030105
Max_low Vs Mean_alpha	0.23212952	0.11372981
Mean_low beta Vs Mean_alpha	0.08636941	0.27344584
Max_low beta Vs Mean_alpha	0.10276058	0.16887104
Mean_high beta Vs Mean_alpha	0.0791078	0.34381258
Max_high beta Vs Mean_alpha	0.07721371	0.25369605
Mean_Beta Vs Mean_alpha	0.22385844	0.62097828
Max_ BetaVs Mean_alpha	0.16594092	0.60219922
Mean_Gamma Vs Mean_alpha	0.15997204	0.0816062
Max_Gamma Vs Mean_alpha	0.07899096	0.49271486

Parameters	Correlation coefficient	P value
Mean_low Vs Max_alpha	0.22060622	0.21147166
Max_low Vs Max_alpha	0.17441103	0.30370137
Mean_low beta Vs Max_alpha	0.12019048	0.37872254
Max_low beta Vs Max_alpha	0.15264991	0.61564219
Mean_high beta Vs Max_alpha	0.13036161	0.30064646
Max_high beta Vs Max_alpha	0.06519334	0.63983148
Mean_Beta Vs Max_alpha	0.09798323	0.23087297
Max_ BetaVs Max_alpha	0.07466379	0.47065925
Mean_Gamma Vs Max_alpha	0.08678156	0.44980331
Max_Gamma Vs Max_alpha	0.09799051	0.37347588

Table 3: The correlation coefficient and P values are listed for the MER, background activity and clinical scores.

Parameters	Correlation coefficient	P value
Mean_low Vs Med_OFF	0.13345341	0.46886331
Max_low Vs Med_OFF	0.05993089	0.44991675
Mean_alpha Vs Med_OFF	0.23054322	0.15687947
Max_alpha Vs Med_OFF	0.23895744	0.12680864
Mean_low beta Vs Med_OFF	0.14817282	0.64944824
Max_low beta Vs Med_OFF	0.58745534	0.00854514*
Mean_high beta Vs Med_OFF	0.14785053	0.15267264
Max_high beta Vs Med_OFF	0.11754388	0.06956049
Mean_Beta Vs Med_OFF	0.23001077	0.38671988
Max_ BetaVs Med_OFF	0.7674564	0.00000521*
Mean_Gamma Vs Med_OFF	0.12384936	0.5791199
Max_Gamma Vs Med_OFF	0.07224055	0.45150518

Parameters	Correlation coefficient	P value
Mean_low Vs Med_ON	0.20605041	0.16425996
Max_low Vs Med_ON	0.12794777	0.27134993
Mean_alpha Vs Med_ON	0.09833826	0.32643556
Max_Alpha Vs Med_ON	0.13078243	0.63898277
Mean_low beta Vs Med_ON	0.06929091	0.14384297
Max_low beta Vs Med_ON	0.67813213	0.00412332*
Mean_high beta Vs Med_ON	0.07639466	0.56331368
Max_high beta Vs Med_ON	0.23841012	0.43685872
Mean_Beta Vs Med_ON	0.24122691	0.27576333
Max_ BetaVs Med_ON	0.60235351	0.00133251*
Mean_Gamma Vs Med_ON	0.16504172	0.16455422
Max_Gamma Vs Med_ON	0.06195591	0.3069518

Parameters	Correlation coefficient	P value
Mean_low Vs LDOPA	0.09695598	0.33921324
Max_low Vs LDOPA	0.12063171	0.12236697
Mean_alpha Vs LDOPA	0.21423881	0.40370449
Max_Alpha Vs LDOPA	0.05308069	0.18571261
Mean_low beta Vs LDOPA	0.05860476	0.28077147
Max_low beta Vs LDOPA	0.08379801	0.39979183
Mean_high beta Vs LDOPA	0.17982309	0.20108367
Max_high beta Vs LDOPA	0.19634448	0.2242644
Mean_Beta Vs LDOPA	0.17954919	0.42025453
Max_ BetaVs LDOPA	0.14018474	0.20916855
Mean_Gamma Vs LDOPA	-0.5794532	0.00243251*
Max_Gamma Vs LDOPA	0.15940178	0.54462576

Parameters	Correlation coefficient	P value
Mean_SR Vs LDOPA	0.10926416	0.63959804
Max_SR Vs LDOPA	0.19893856	0.48814928
ISI Vs LDOPA	0.087791	0.2563262
BI Vs LDOPA	0.18735509	0.4004416
LvR Vs LDOPA	0.08670223	0.11466141
Mean RMS Vs LDOPA	0.12369692	0.59378489
Max RMS Vs LDOPA	0.17512371	0.57779223

Parameters	Correlation coefficient	P value
Mean_SR Vs Med_ON	0.20604549	0.54065634
Max_SR Vs Med_ON	0.06622515	0.2064368
ISI Vs Med_ON	0.23587719	0.40661375
BI Vs Med_ON	0.20514254	0.06350756
LvR Vs Med_ON	0.14735833	0.30515559
Mean RMS Vs Med_ON	0.13717172	0.23763133
Max RMS Vs Med_ON	0.13935675	0.14689085

Parameters	Correlation coefficient	P value
Mean_SR Vs Med_OFF	0.11126989	0.15725971
Max_SR Vs Med_OFF	0.15170173	0.30373141
ISI Vs Med_OFF	0.15215431	0.1065376
BI Vs Med_OFF	0.21352554	0.4091142
LvR Vs Med_OFF	0.20896628	0.33255455
Mean RMS Vs Med_OFF	0.17886363	0.46756959
Max RMS Vs Med_OFF	0.12572188	0.46993271
